# A System of Clinical Medicine

**Published:** 1849

**Authors:** 


ARTICLE X.

*A System of Clinical Medicine.* By ROBERT JAMES GRAVES, M. D., one of the Physicians of the Meath Hospital and County of Dublin Infirmary; formerly Professor of the Institutes of Medicine, &c., &c. With notes and a series of lectures, by W. W. GERHARD, M. D., Lecturer on Clinical Medicine to the University of Pennsylvania, one of the Physicians to the Pennsylvania Hospital, &c., &c. Third American Edition. pp. 751 8vo. Philadelphia: Ed. Barrington & Geo. D. Haswell, 1848. (From the Publishers—for sale at Keenes', Chicago.)

This work stands decidedly approved by the American Medical Public. Clinical Medicine is, in fact a very important feature of the subject of practice; and as this work occupies the field almost alone, it is not surprising that it should be in such active demand, as to require this new edition.

Thls edition is made from the recent and much enlarged European edition of Dr. Graves' lectures.

We can most heartily recommend the work before us as one containing a large amount of the best information on the subject of practice in many of the diseases prevalent in Europe and America. E.

